# Supplementary material for: Synaptic vesicle proteins and ATG9A self-organize in distinct vesicle phases within synapsin condensates
Source: Nat Commun. 2023 Jan 28;14:455. doi: 10.1038/s41467-023-36081-3 (PMC9884207; doi:10.1038/s41467-023-36081-3)
Supplement: Supplementary file 1 — Supplementary Information [file 41467_2023_36081_MOESM1_ESM.pdf]

Supplementary Information for

Synaptic vesicle proteins and ATG9A self-organize in distinct vesicle phases within synapsin condensates

Daehun Park[1-4], Yumei Wu[1-4], Xinbo Wang[1-4], Swetha Gowrishankar[5], Aaron Baublis[6] and Pietro De Camilli[1-4,7]

[1]Department of Neuroscience, Yale University School of Medicine, New Haven, Connecticut 06510, USA.
[2]Department of Cell Biology, Yale University School of Medicine, New Haven, Connecticut 06510, USA.
[3]Howard Hughes Medical Institute, Yale University School of Medicine, New Haven, Connecticut 06510, USA.
[4]Program in Cellular Neuroscience, Neurodegeneration, and Repair, Yale University School of Medicine, New Haven, Connecticut 06510, USA.
[5]Department of Anatomy and Cell Biology, College of Medicine, University of Illinois at Chicago, Chicago, Illinois 60612, USA.
[6]Harvard Chan Advanced Multi-omics Platform, Harvard T.H. Chan School of Public Health, Boston, Massachusetts 02115, USA.
[7]Kavli Institute for Neuroscience, Yale University School of Medicine, New Haven, Connecticut 06510, USA.

Corresponding author: Pietro De Camilli (pietro.decamilli@yale.edu)

This PDF file includes:

Supplementary Figures 1-10

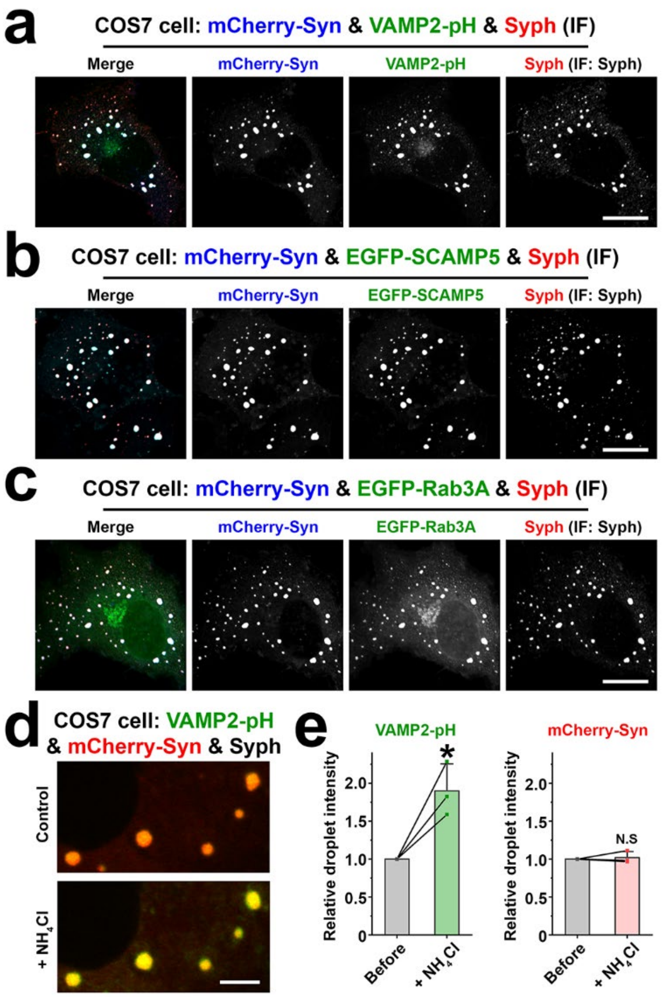

Supplementary Fig. 1 SV proteins coassemble into droplets formed by synaptophysin and synapsin. a-c, COS7 cells were transfected as indicated and synaptophysin was detected by immunofluorescence using anti-synaptophysin antibodies. d, COS7 cell expressing synaptophysin, VAMP2-pH and mCherry-synapsin before and after treatment with NH_4Cl to alkalinize the acidic lumen of the vesicles and thus increase pHluorin fluorescence. e, Quantification of the fluorescence changes after the addition of NH_4Cl. Values are means ± SD; N.S., not significant; *$p < 0.05$ by two-sided paired t-test (72 droplets from 3 independent treatments were quantified). Source data are provided as a Source Data file. Scale bars, a-c = 20 µm, d = 5 µm. p values (e): 4.835×10^{-2} (VAMP2), 0.7103 (mCherry-Syn).

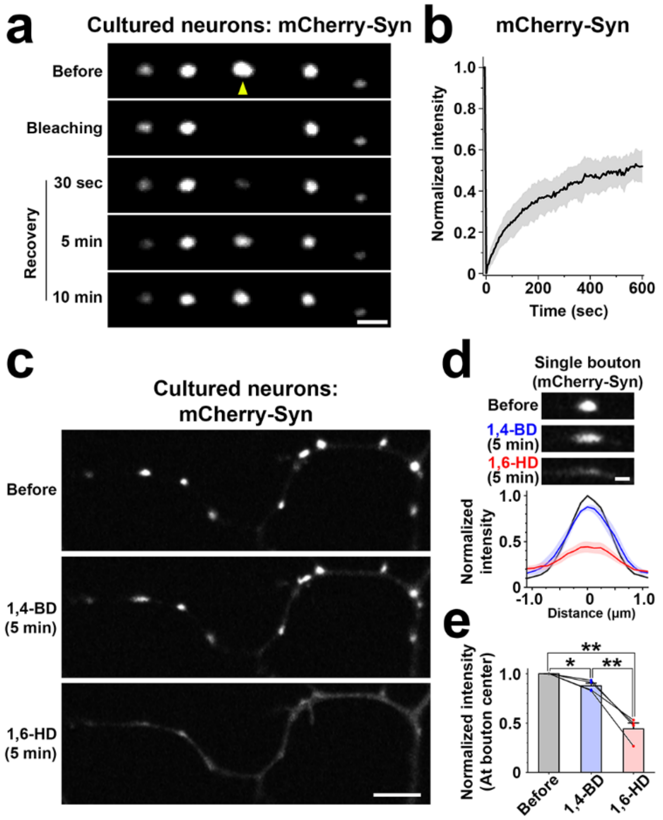

Supplementary Fig. 2 Liquid-like properties of synapsin condensates in cultured neurons. a, Representative time-lapse images showing fluorescence recovery of mCherry-synapsin after photobleaching of a single bouton. b, A Plot of the average fluorescence intensities after photobleaching of four mCherry-synapsin positive boutons. Data are represented as mean ± SD. c, Live fluorescence imaging of hippocampal neuronal cultures expressing mCherry-synapsin. Neurons were treated with 3% 1,4-Butanediol (1,4-BD) for 5 min. After 5 min washout, the same neurons were treated with 3% 1,6-Hexanediol (1,6-HD) for 5 min. d, mCherry-synapsin intensity on a presynaptic bouton and flanking axonal regions before and 5 min after addition of 1,4-Butanediol or 1,6-Hexanediol. Fluorescent images are shown at the top. e, Statistical comparison of mCherry-synapsin fluorescence intensities at each bouton centers. Values are means ± SEM of 4 independent experiments (total 18 boutons and flanking axonal regions were analyzed). *$p < 0.05$; **$p < 0.01$. Two-sided paired t-tests were performed on significant one-way repeated measures ANOVA. Source data are provided as a Source Data file. Scale bars, a = 2 µm, c = 5 µm, d = 1 µm. p values (e): 1.961×10^{-2} (Before vs 1,4-BD), 2.611×10^{-3} (Before vs 1,6-HD), 3.815×10^{-3} (1,4-BD vs 1,6-HD).

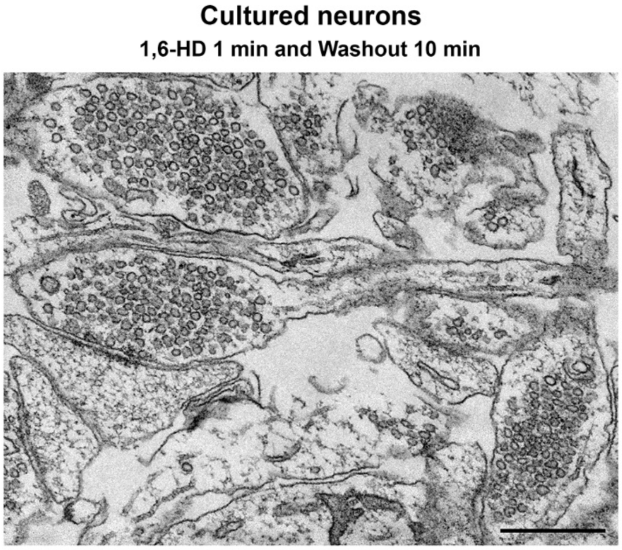

Supplementary Fig. 3 Reversible dispersion of SV clusters by 1,6-Hexanediol in nerve terminals. Cultured hippocampal neurons were washed for 10 min after 1 min incubation with 1,6-Hexanediol and then fixed for transmission electron microscopy (TEM). Scale bar = 500 nm.

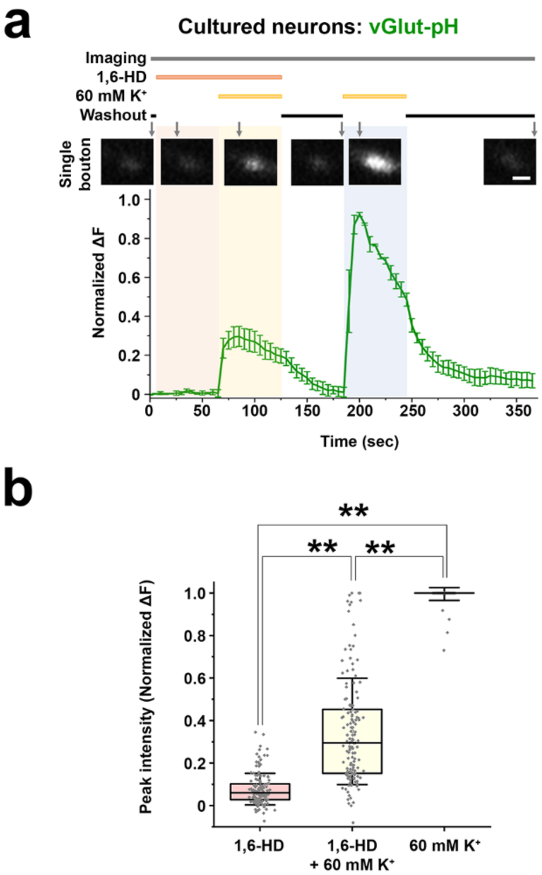

Supplementary Fig. 4 1,6-Hexanediol inhibits synaptic vesicle release in cultured neurons. a, Normalized average vGlut-pHluorin fluorescence intensity profile and representative time lapse images of presynaptic boutons from cultured hippocampal neurons stimulated with high K^+ in the presence or absence of 1,6-Hexanediol. See also Supplementary Video 3. Values are means ± SEM of 3 independent experiments (total 143 boutons were analyzed). b, Peak vGlut-pHluorin fluorescence intensity values of presynaptic boutons in neurons exposed to 1,6-Hexanediol before and during a first high K^+ stimulation and then during a second high K^+ stimulation in the absence of 1,6-Hexanediol. Box plots show the median line (midline), 25/75 percentiles (boxes), and SD (whiskers) at each condition. $^{**}p < 0.01$ by one-way ANOVA with Tukey's HSD post hoc test (143 boutons from 3 independent experiments were analyzed). Source data are provided as a Source Data file. Scale bar, a = 1 µm. p values (e): 5.880×10^{-3} (1,6-HD vs 1,6-HD + 60 mM K^+), 1.272×10^{-5} (1,6-HD vs 60 mM K^+), 1.287×10^{-4} (1,6-HD + 60 mM K^+ vs 60 mM K^+).

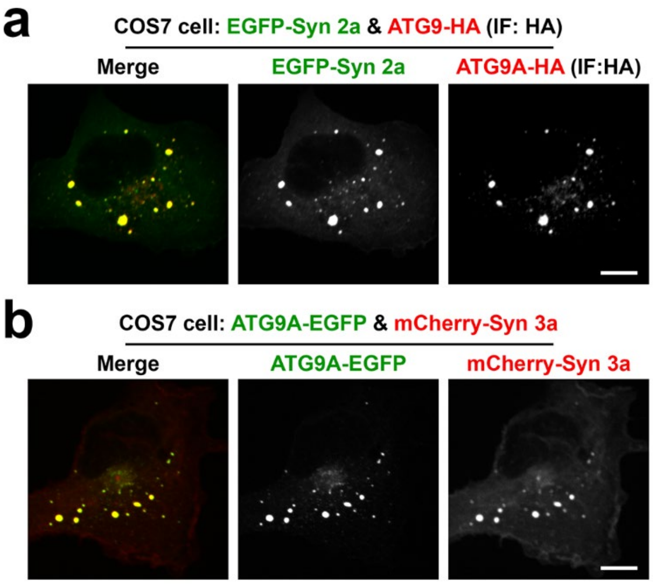

Supplementary Fig. 5 Synapsin isoforms, synapsin 2a and 3a, form droplets with ATG9A. a, COS7 cells expressing ATG9A-HA and EGFP-synapsin 2a (EGFP-Syn 2a) were fixed and stained with HA antibodies. b, ATG9A-EGFP and mCherry-synapsin 3a (mCherry-Syn 3a) co-expressing COS7 cells were fixed and imaged. Scale bar = 10 µm.

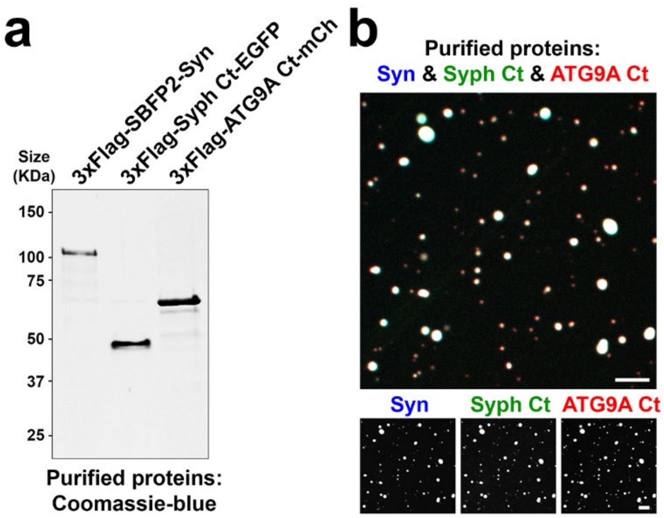

Supplementary Fig. 6 Synapsin, synaptophysin Ct and ATG9A Ct assemble into the same droplets *in vitro*. a, N-terminal 3xFlag conjugated SBFP2-synapsin (full length), synaptophysin Ct (cytosolic C-terminal region only: 219-308 aa)-EGFP and ATG9A Ct (C-terminal region only: 523-839 aa)-mCherry were purified from Expi293 cells and analyzed by SDS-PAGE and Coomassie Blue staining. b, Co-assembly of synapsin, synaptophysin Ct and ATG9A Ct into liquid droplets. The three purified proteins were mixed in a buffer containing 100 mM NaCl, 25mM Tris-HCl (pH 7.4), 0.5mM TCEP and 7% PEG. Source data are provided as a Source Data file. Scale bars = 5 μm.

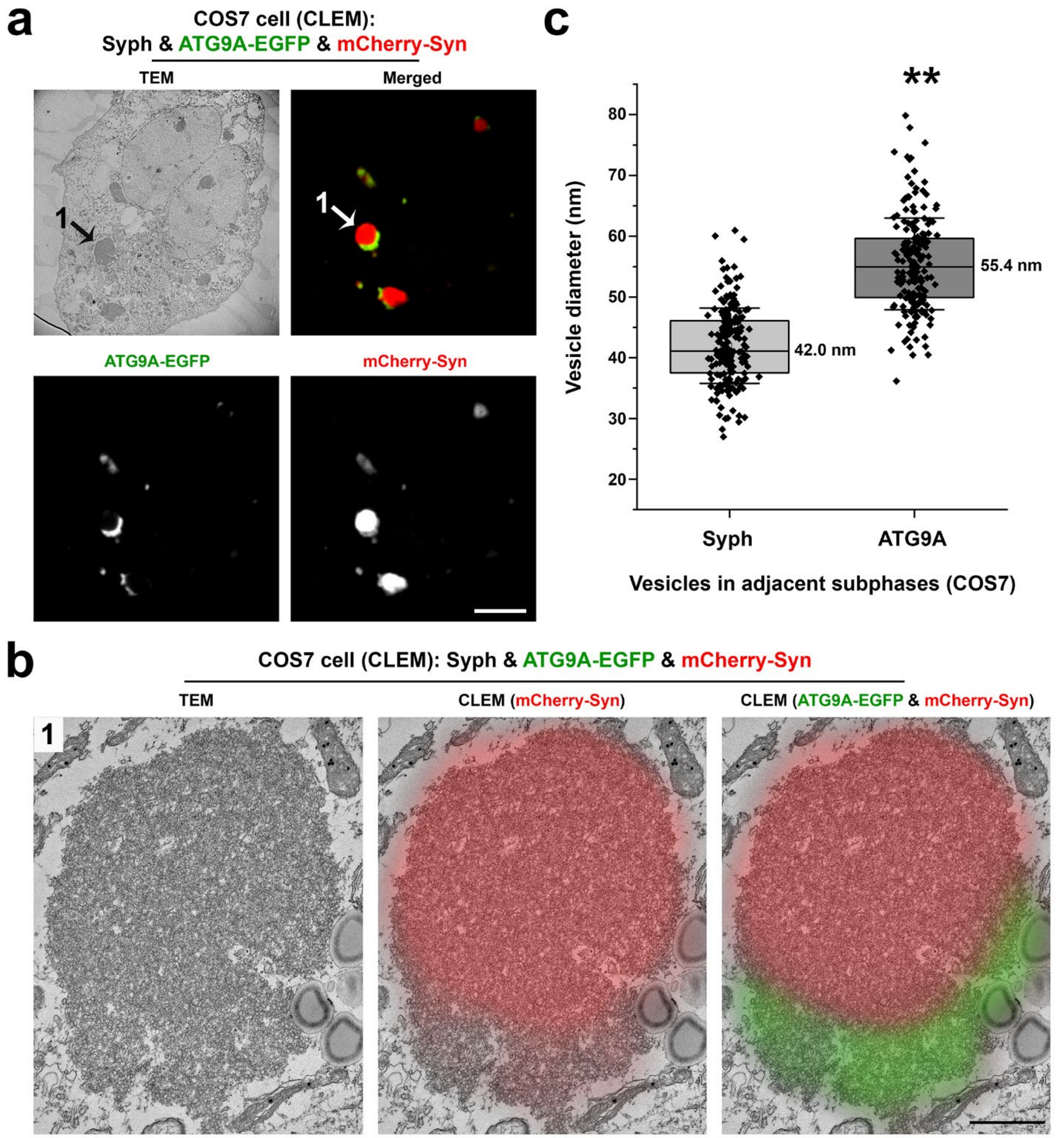

Supplementary Fig. 7 The synapsin phases include two distinct vesicle clusters positive for synaptophysin and ATG9A, respectively. a, COS7 cells co-expressing synaptophysin, ATG9A-EGFP and mCherry-synapsin were fixed and processed for correlative light-electron microscopy (CLEM). b, High magnification of fluorescence and TEM image of droplet #1. c, Size distribution of the ATG9A and synaptophysin vesicles when the two vesicles make subphases within a synapsin phase. Box plots show the median line (midline), 25/75 percentiles (boxes), and SD (whiskers). Data are represented as mean. 200 vesicles were measured for each group (from four different EM images). $**p < 0.01$, by two-sided Student's t-test. Source data are provided as a Source Data file. Scale bars, a = 10 μm, b = 500 nm. p value (c): 6.455×10^{-6}.

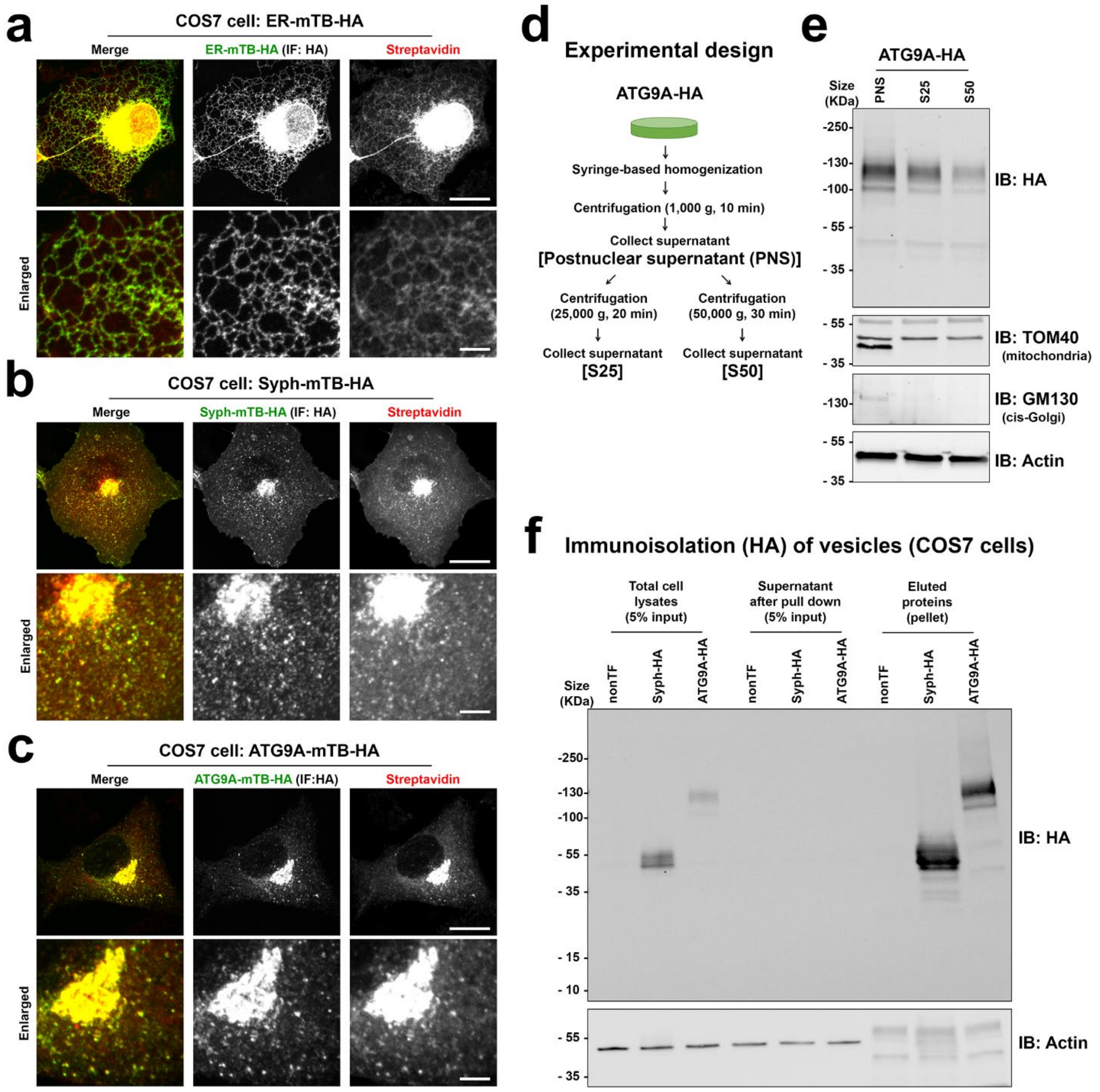

Supplementary Fig. 8 Control experiments for proteomic analysis. a-c, COS7 cells expressing ER-miniTurboID-HA (a), synaptophysin-miniTurboID-HA (b) or ATG9A-miniTurboID-HA (c) were treated with 500 µM biotin for 15 min at 37°C, then fixed and processed for anti-HA immunofluorescence or streptavidin labeling. d, Experimental design for vesicle immunoisolation. e, Preparation of vesicle-enriched fraction for vesicle immunoisolation. The postnuclear supernatant and the supernatants resulting from two different centrifugations were analyzed by Western blotting for the indicated proteins. The S25 supernatant was chosen for the immunoisolation given its loss of Golgi and mitochondrial markers and retention of the bulk of small vesicles (ATG9A) as detected by Western blotting. f, Starting S25 fraction, and material bound and not bound by anti-HA magnetic beads were analyzed by Western blotting. Source data are provided as a Source Data file. Scale bars, a-c = 20 µm (5 µm for the enlarged images).

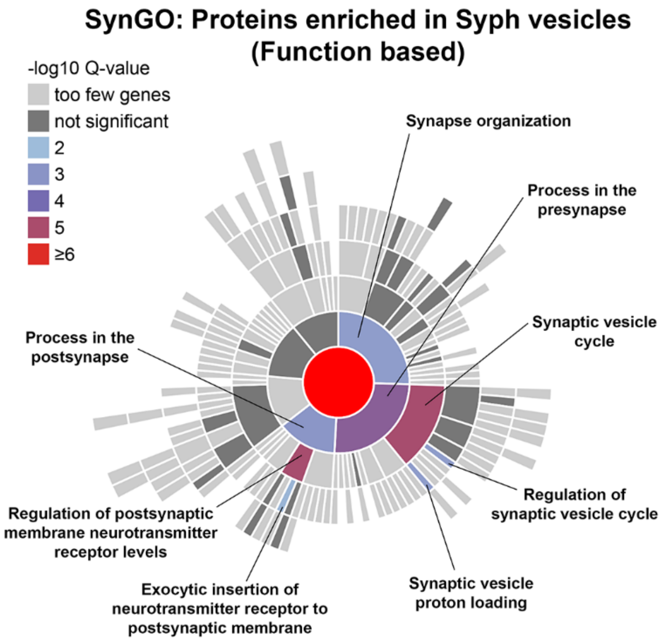

Supplementary Fig. 9 SynGO analysis of proteins enriched in synaptophysin-HA immunoisolated samples. Sunburst plot showing synaptic function annotations of the proteins enriched in synaptophysin-HA versus ATG9A-HA immunoisolated fractions. Inner rings are parent terms of more specific child terms in the outer rings. Colors represent enrichment Q value (-log10 values).

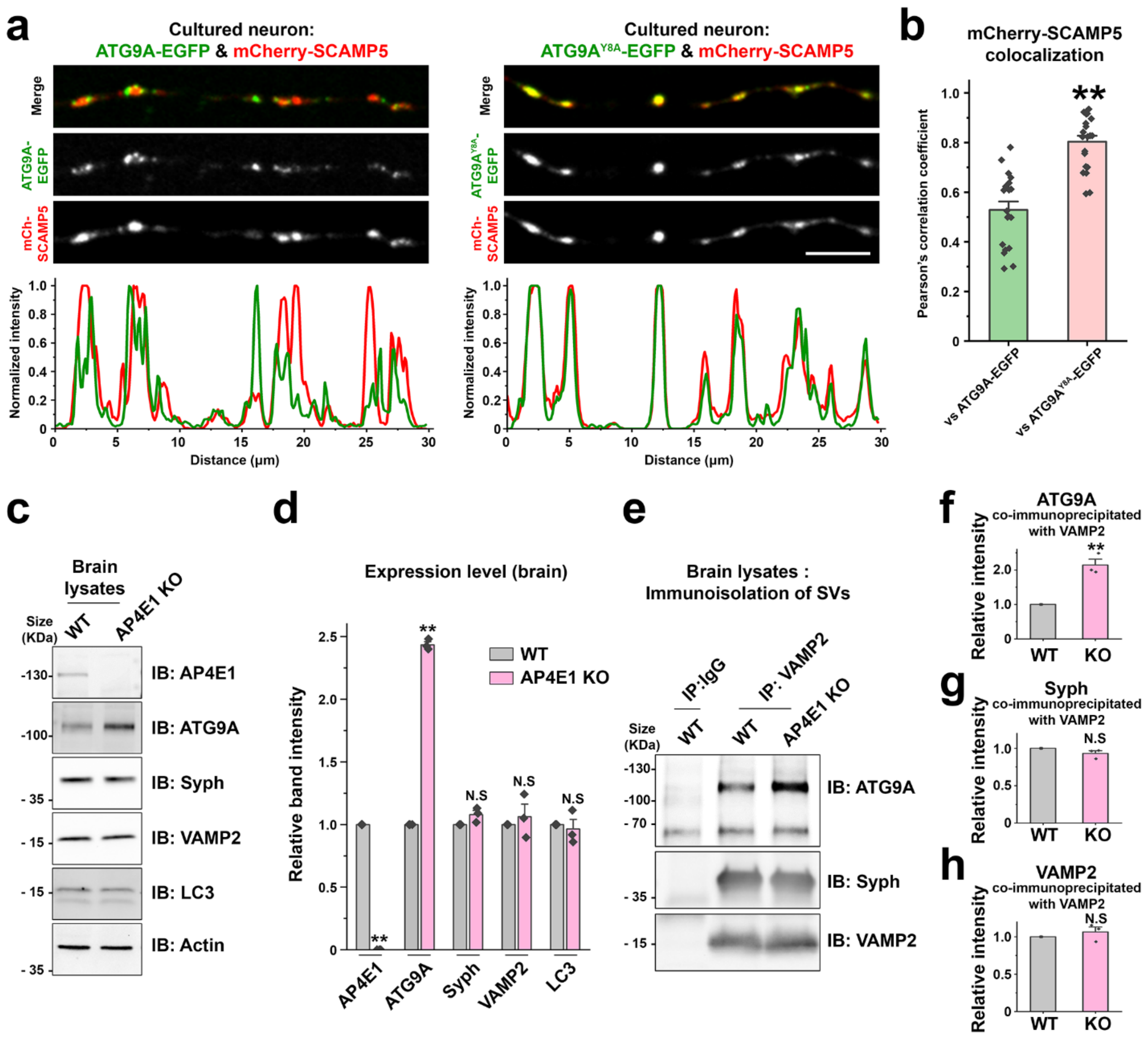

Supplementary Fig. 10 Importance of AP4 binding for proper sorting and segregation of ATG9A vesicles in neurons. a, Axonal varicosities of an axon expressing mCherry-SCAMP5 with either ATG9A-EGFP or ATG9A^{Y8A}-EGFP. Corresponding line-scans are shown at the bottom. b, Colocalization analysis. Values are means ± SEM. ***p* < 0.01 by two-sided Student's t-test (all boutons in 19 (WT) or 21 (Y8A) field of view (121 μm x 121 μm) from three independent cultures were quantified). c,d, Representative Western blot images and quantification (n = 3 independent blots) of the expression levels of several proteins in AP4E1 knockout (AP4E1 KO) mouse brains (8-month-old). Littermate wildtype mouse brains were used as a control (WT). ***p* < 0.01; N.S., not significant by two-sided Student's t-test. e, Synaptic vesicles were immunoisolated from either WT or AP4E1 KO brains using anti-VAMP2 antibody conjugated magnetic beads and immunoblotted with ATG9A, synaptophysin and VAMP2 antibodies. f-h, Quantifications from three independent blots. Values are means ± SEM. ***p* < 0.01; N.S., not significant by two-sided Student's t-test. Source data are provided as a Source Data file. Scale bar = 5 μm. p values (b): 5.280×10^{-8}, (d): 0 (AP4E1), 4.689×10^{-7} (ATG9A), 0.0748 (Syph), 0.5627 (VAMP2), 0.6665 (LC3). (f): 2.685×10^{-3}. (g): 0.1276. (h): 0.3852.
