# Supplementary material for: Synaptic vesicle proteins and ATG9A self-organize in distinct vesicle phases within synapsin condensates
Source: Nat Commun. 2023 Jan 28;14:455. doi: 10.1038/s41467-023-36081-3 (PMC9884207; doi:10.1038/s41467-023-36081-3)
Supplement: Supplementary file 2 — Description of Additional Supplementary Files [file 41467_2023_36081_MOESM2_ESM.pdf]

Description of Additional Supplementary Files

Supplementary Data 1. Full proteomic datasets for proximity biotinylation and immunoisolation of synaptophysin and ATG9A vesicles.

Supplementary Video 1. 1,6-Hexanediol (1,6-HD) dependent reversible dispersion of the condensates formed by synaptophysin, VAMP2-pH (green) and mCherry-synapsin (red) co-expression. Related to Fig. 2e.

Supplementary Video 2. 1,6-Hexanediol (1,6-HD) dependent reversible dispersion of the SV clusters in cultured neurons. Neurons were co-transfected with EGFP-SCAMP5 (an integral SV membrane protein, green) and mCherry-synapsin (a SV associated protein, red). Related to Fig. 2f-i.

Supplementary Video 3. SV exocytosis, as reflected by the increase in vGlut-pHluorin fluorescence, in the presence or absence of 1,6-Hexanediol (1,6-HD). vGlut-pHluorin and mCherry-synapsin expressing neurons were treated as indicated. Related to Fig. 2k,l and Extended Data Fig. 4.

Supplementary Video 4. 1,6-Hexanediol (1,6-HD) dependent reversible dispersion of two distinct vesicle clusters formed by synaptophysin, ATG9A-EGFP and mCherry-synapsin coexpression in COS7 cells. Related to Fig. 4f.

Supplementary Video 5. Z stack images of COS7 cells expressing $ATG9A^{Y8A}$. The cis-Golgi was detected by immunofluorescence using GM130 antibodies. 0.2 μm / each Z sections (total 42 images). Related to Fig. 7b.
