# Supplementary material for: Synaptic vesicle proteins and ATG9A self-organize in distinct vesicle phases within synapsin condensates
Source: Nat Commun. 2023 Jan 28;14:455. doi: 10.1038/s41467-023-36081-3 (PMC9884207; doi:10.1038/s41467-023-36081-3)
Supplement: Supplementary file 9 — Reporting Summary [file 41467_2023_36081_MOESM9_ESM.pdf]

nature portfolio

Corresponding author(s): Pietro De Camilli

Last updated by author(s): Jan 3, 2022

Reporting Summary

Nature Portfolio wishes to improve the reproducibility of the work that we publish. This form provides structure for consistency and transparency in reporting. For further information on Nature Portfolio policies, see our Editorial Policies and the Editorial Policy Checklist.

Statistics

For all statistical analyses, confirm that the following items are present in the figure legend, table legend, main text, or Methods section.

n/a	Confirmed	
☐	☒	The exact sample size (*n*) for each experimental group/condition, given as a discrete number and unit of measurement
☐	☒	A statement on whether measurements were taken from distinct samples or whether the same sample was measured repeatedly
☐	☒	The statistical test(s) used AND whether they are one- or two-sided<br>*Only common tests should be described solely by name; describe more complex techniques in the Methods section.*
☒	☐	A description of all covariates tested
☐	☒	A description of any assumptions or corrections, such as tests of normality and adjustment for multiple comparisons
☐	☒	A full description of the statistical parameters including central tendency (e.g. means) or other basic estimates (e.g. regression coefficient) AND variation (e.g. standard deviation) or associated estimates of uncertainty (e.g. confidence intervals)
☐	☒	For null hypothesis testing, the test statistic (e.g. *F*, *t*, *r*) with confidence intervals, effect sizes, degrees of freedom and *P* value noted<br>*Give P values as exact values whenever suitable.*
☒	☐	For Bayesian analysis, information on the choice of priors and Markov chain Monte Carlo settings
☒	☐	For hierarchical and complex designs, identification of the appropriate level for tests and full reporting of outcomes
☐	☒	Estimates of effect sizes (e.g. Cohen's *d*, Pearson's *r*), indicating how they were calculated

Our web collection on statistics for biologists contains articles on many of the points above.

Software and code

Policy information about availability of computer code

Data collection	UltraVIEW (PerkinElmer) and Velox software (Thermo Fisher Scientific)
Data analysis	ImageJ 1.53q, Origin 2022b, SpectronautTM software version 15 (Biognosys AG) and SigmaPlot version 10.0 (Systat).

For manuscripts utilizing custom algorithms or software that are central to the research but not yet described in published literature, software must be made available to editors and reviewers. We strongly encourage code deposition in a community repository (e.g. GitHub). See the Nature Portfolio guidelines for submitting code & software for further information.

Data

Policy information about availability of data

All manuscripts must include a data availability statement. This statement should provide the following information, where applicable:

- Accession codes, unique identifiers, or web links for publicly available datasets
- A description of any restrictions on data availability
- For clinical datasets or third party data, please ensure that the statement adheres to our policy

Data supporting the findings of this study are available in the Paper, Supplementary Information, Supplementary Movies and Source Data, and are also available from the corresponding author upon request. The mass spectrometry proteomics data have been deposited to the ProteomeXchange Consortium via the PRIDE partner repository with the dataset identifier PXD037490. Source data are provided with this paper.

Human research participants

Policy information about studies involving human research participants and Sex and Gender in Research.

Reporting on sex and gender	N/A
Population characteristics	N/A
Recruitment	N/A
Ethics oversight	N/A

Note that full information on the approval of the study protocol must also be provided in the manuscript.

Field-specific reporting

Please select the one below that is the best fit for your research. If you are not sure, read the appropriate sections before making your selection.

- [x] Life sciences
- [ ] Behavioural & social sciences
- [ ] Ecological, evolutionary & environmental sciences

For a reference copy of the document with all sections, see nature.com/documents/nr-reporting-summary-flat.pdf

Life sciences study design

All studies must disclose on these points even when the disclosure is negative.

Sample size	No statistical methods were used to predetermine sample size. Where required for statistical analyses, sample sizes were determined based on established practice and according to accepted standards in this field: Biological triplicates (at least 3 independent experiments).
Data exclusions	No data were excluded.
Replication	All representative light microscopy images and Western blot images were obtained from at least three independent experiments with similar results. CLEM experiments and in vitro assay were performed twice.
Randomization	Although intentional randomization is not relevant for this study, a sufficient number of cells were examined for a given experiment and at the time of experiments, cells were taken out of incubator randomly thus randomization of cells with respect to treatment is inherent to our experimental design.
Blinding	Investigators were not blinded to group allocation during data collection and analysis, because experiments were frequently done by the same investigators.

Reporting for specific materials, systems and methods

We require information from authors about some types of materials, experimental systems and methods used in many studies. Here, indicate whether each material, system or method listed is relevant to your study. If you are not sure if a list item applies to your research, read the appropriate section before selecting a response.

Materials & experimental systems

n/a	Involved in the study	
[ ]	[x]	Antibodies
[ ]	[x]	Eukaryotic cell lines
[x]	[ ]	Palaeontology and archaeology
[ ]	[x]	Animals and other organisms
[x]	[ ]	Clinical data
[x]	[ ]	Dual use research of concern

Methods

n/a	Involved in the study	
[x]	[ ]	ChIP-seq
[x]	[ ]	Flow cytometry
[x]	[ ]	MRI-based neuroimaging

Antibodies

Antibodies used	Primary antibodies:<br>anti-synaptophysin (101 002, Synaptic Systems)<br>anti-synaptophysin (101 011, Synaptic Systems)<br>anti-synaptophysin (101 308, Synaptic Systems)

anti-VAMP2 (104 211, Synaptic Systems)
anti-Rab3A (107 111, Synaptic Systems)
anti-ATG9A (ab108338, Abcam)
anti-LC3 (PM036, MBL)
anti-AP4E1 (612018, BD Transduction Laboratories™)
anti-Actin (691002, MP Biomedicals)
anti-HA (MMS-101R, Covance)
anti-GM130 (610822, BD Bioscience)
anti-TGN46 (610822, AHP500GT, AbD Serotec)
anti-TOMM40 (18409-1-AP, Proteintech)

Materials for IF:
Alexa Fluor 488 anti-mouse IgG (H+L) (A11001, Invitrogen)
Alexa Fluor 594 anti-mouse IgG (H+L) (A21203, Invitrogen)
Alexa Fluor 647 anti-mouse IgG (H+L) (A32787, Invitrogen)
Alexa Fluor 594 anti-rabbit IgG (H+L) (A11037, Invitrogen)
Alexa Fluor 647 anti-sheep IgG (H+L) (A21448, Invitrogen)
Alexa Fluor 488 anti-guinea pig IgG (H+L) (A11073, Invitrogen)
Streptavidin, Alexa Fluor 647 Conjugate (S32357, Invitrogen)

Materials for WB:
IRDye 800CW anti-mouse (926-32210, LI-COR)
IRDye 800CW anti-rabbit (926-32211, LI-COR)
IRDye 800CW anti-guinea pig (925-32411, LI-COR)
IRDye 680LT anti-mouse (926-68020, LI-COR)
IRDye 680LT anti-rabbit (926-68021, LI-COR)
IRDye 680RD Streptavidin (926-68079, LI-COR)

Validation

anti-synaptophysin (101 002): Suitable for WB, IP, ICC, IHC, EM, ELISA; reacts with Human, Rat, Mouse, Hamster, Cow, Chicken, Frog, Zebrafish [https://sysy.com/product/101002]

anti-synaptophysin (101 011): Suitable for WB, IP, ICC, IHC, IHC-P, EM, ELISA; reacts with Human, Rat, Mouse, Mammals (weak signals for Zebrafish and other vertebrates. Other species not tested yet); KO validated PubMed: 31940485 [https://sysy.com/product/101011]

anti-synaptophysin (101 308): Suitable for WB, IP, ICC, IHC, IHC-P; reacts with Human, Rat, Mouse, Mammals (weak signals for Zebrafish and other vertebrates. Other species not tested yet); KO validated [https://sysy.com/product/101308]

anti-VAMP2: Suitable for WB, IP, ICC, IHC, IHC-P, EM, ELISA; reacts with Human, Rat, Mouse, Hamster (No signal: Chicken and Zebrafish. Other species not tested yet); KO validated PubMed: 26663078 [https://sysy.com/product/104211]

anti-Rab3A: Suitable for WB, IP, ICC, IHC, IHC-P, EM, ELISA; reacts with Human, Rat, Mouse, Mammals (Other species not tested yet); Specific for mammalian Rab 3a; KO validated PubMed: 12244319 [https://sysy.com/product/107111]

anti-ATG9A: Suitable for Flow Cyt (Intra), WB, IP, IHC-P, ICC/IF; reacts with Human, Rat, Mouse; KO validated [https://www.abcam.com/atg9a-antibody-epr24502-ab108338.html]

anti-LC3: Suitable for FC, ICC, IHC, IP, WB; reacts with Human, Rat, Mouse, Hamster [https://www.mblintl.com/products/pm036/]

anti-AP4E1: Suitable for WB (IF: Not recommended); reacts with Human [https://www.bdbiosciences.com/en-us/products/reagents/western-blotting-and-molecular-reagents/western-blot-reagents/purified-mouse-anti-adaptin.612018]

anti-Actin: Suitable for ELISA, IHC, WB, ICC/IF; reacts with Human, Rat, Mouse, Guinea Pig [https://www.mpbio.com/us/anti-actin-mouse-monoclonal-antibody-clone-c4]

anti-HA: Suitable for WB, ICC, IP, FC, Purification; This antibody was raised against the twelve amino acid peptide CYPYDVPDYASL [https://www.biolegend.com/fr-ch/products/anti-ha-11-epitope-tag-antibody-11071]

anti-GM130: Suitable for WB, IF IHC, IP (Not recommended); reacts with Human, Rat, Mouse, Dog [https://www.bdbiosciences.com/en-nz/products/reagents/microscopy-imaging-reagents/immunofluorescence-reagents/purified-mouse-anti-gm130.610822]

anti-TGN46: Suitable for WB, IF IHC; reacts with Human (cross-reactivity to Primate) [https://www.bio-rad-antibodies.com/polyclonal/human-tgn46-antibody-ahp500.html?f=purified&JSESSIONID_STERLING=CC593AAC0B8559394F5A8FF87D38F07B.ecommerce1&evCntryLang=US-en&cntry=US&thirdPartyCookieEnabled=true]

anti-TOMM40: Suitable for WB, IP, IHC, IF, FC, CoIP, ELISA; reacts with Human, Rat, Mouse, Bovine, Monkey [https://www.ptglab.com/products/TOMM40-Antibody-18409-1-AP.htm]

Eukaryotic cell lines

Policy information about cell lines and Sex and Gender in Research

Cell line source(s)	COS7 cells were purchased from ATCC.<br>Expi293F cells were purchased from Thermo Fisher Scientific
Authentication	This lines were not authenticated.
Mycoplasma contamination	We routinely test our cells for mycoplasma: All cell lines tested negative for mycoplasma contamination.
Commonly misidentified lines (See ICLAC register)	No commonly misidentified cell lines were used.

Animals and other research organisms

Policy information about studies involving animals; ARRIVE guidelines recommended for reporting animal research, and Sex and Gender in Research

Laboratory animals	Post-natal day 0 C57BL/6 (B6) wild type mouse pups of either sex were used for dissociated hippocampal cultures.<br>C57BL/6 (B6) mice with a deletion of exon 3 of the AP4E1 gene (AP4E1KO) and their litter-mate controls (wild type) were sacrificed to collect the brain samples (sex: female, age: 8-month-old).<br>Mouse colony was maintained in the vivarium with a 12 hour light/dark cycle, stable temperature at 22°C ± 1°C, and humidity between 20 and 50%.
Wild animals	This study did not involve wild animals.
Reporting on sex	Sex was not considered in our study design as it was not deemed to be relevant to the questions addressed.
Field-collected samples	This study did not include field-collected samples.
Ethics oversight	Institutional Animal Care and Use Committees of Yale University.

Note that full information on the approval of the study protocol must also be provided in the manuscript.
